# When the Trial Ends: The Case for Post-Trial Provisions in Clinical Psychedelic Research

**DOI:** 10.1007/s12152-023-09536-z

**Published:** 2023-11-06

**Authors:** Edward Jacobs, Ashleigh Murphy-Beiner, Ian Rouiller, David Nutt, Meg J. Spriggs

**Affiliations:** 1 Department of Psychiatry, University of Oxford, Oxford, UK; 2 Wellcome Centre for Ethics and Humanities, University of Oxford, Oxford, UK; 3 Department of Brain Sciences, Imperial College London, London, UK; 4 Department of Psychology, Royal Holloway University, London, UK; 5 Psychedelic Participant Advocacy Network (PsyPAN), London, UK

**Keywords:** Research ethics, Psychedelic, Psilocybin, Clinical trials, Post-trial access

## Abstract

The ethical value—and to some scholars, necessity—of providing trial patients with post-trial access (PTA) to an investigational drug has been subject to significant attention in the field of research ethics. Although no consensus has emerged, it seems clear that, in some trial contexts, various factors make PTA particularly appropriate. We outline the atypical aspects of psychedelic clinical trials that support the case for introducing the provision of PTA within research in this field, including the broader legal status of psychedelics, the nature of the researcher-therapist/participant relationship, and the extended time-frame of the full therapeutic process. As is increasingly understood, the efficacy of psychedelic-assisted psychotherapy is driven as much by extrapharmacological elements and the cultural therapeutic container as by the drug itself. As such, we also advocate for a refocusing of attention from post-trial access to a broader concept encompassing other elements of post-trial care. We provide an overview of some of the potential post-trial care provisions that may be appropriate in psychedelic clinical trials. Although the World Medical Association’s Declaration of Helsinki calls on researchers, sponsors, and governments to make provisions for post-trial access, such provision may feel impracticable or out-of-reach within psychedelic trials that are already constrained by a high resource demand and significant bureaucratic burden. We show how conceiving of post-trial provision as an integral site of the research process, and an appropriate destination for research funding, will serve to develop the infrastructure necessary for the post-legalisation psychedelic medicine ecosystem.

## Introduction

The World Medical Association’s Declaration of Helsinki [1], a guiding statement of ethical principles for clinicians undertaking medical research, is widely relied upon as a benchmark for ethical practice in clinical research, and is often cited in reports of psychedelic clinical trials. Originally developed in 1964 with the goal of protecting human participants during the course of clinical research trials, it has survived nine revisions, the most recent of which broadened focus to protecting human participants both during trials, and after a trial has been completed. The 2013 revision calls on the sponsors, researchers, and host governments of clinical trials to “make provisions for post-trial access for all participants who still need an intervention identified as beneficial during the study.” Put another way, the declaration calls for study providers to offer continued access to favorable treatments beyond the time-limited clinical trial. Although laudable in its aims, the matter of post-trial provisions is a significantly contested space [2, 3]. Even if all three of the actors called upon in the declaration – sponsors, researchers, and host governments – are sympathetic to the aim, the declaration is quiet on the matter of how the responsibility should be divided, and a responsibility that is vague is thereby less likely to be discharged [4]. Moreover, relevant national and international frameworks, though they can agree on the need for post-trial provisions, disagree both over the strength and nature of the obligations involved, and, differ in which (if any) specific stakeholders are required to act [5, 6]. For example, the Council for International Organizations of Medical Science (CIOMS), established by the World Health Organization and the United Nations Educational, Scientific and Cultural Organization, recognizes post-trial responsibilities on the part of researchers and sponsors, but provides ambiguous guidance on the criteria for provision [7].

The extent of the required post-trial provisions, and the weight of the moral obligation to see they are delivered, are understood to vary between trials depending on contextual factors (see [2, 3]). For example, undertaking clinical research to develop pharmaceuticals in low resource countries where the same drugs will be subsequently unaffordable risks exploitation [8]. However, there remain questions over what grounds these responsibilities, who holds them, and for how long [4, 5, 9], and what an acceptable execution of these responsibilities looks like. In the current article, we do not seek to present a substantive, generalizable normative principle for determining what responsibilities exist across all clinical trials. Rather, we aim to 1) draw attention to broad ethical considerations specific to psychedelic medicines that support the expansion of post-trial provision in clinical trials, 2) begin to identify a range of post-trial *care* practices to consider that includes, but is not limited to, post-trial *access* to psychedelic medicines, in order to meet the obligations towards participants, and 3) discuss how such an expansion is in the longer-term interests of the clinical psychedelic community, and how it fits within the wider healthcare ecosystem required for the successful mainstreaming of psychedelic medicines.

We begin by outlining the nature of psychedelic-assisted therapy, the hybrid pharmacotherapy-psychotherapy modality which is used in almost all modern psychedelic clinical trials. It is the atypical features of this modality, compared to standard drug trials, which drive much of the ethical case for post-trial provisions. Next, we describe an exemplar candidate for post-trial access to additional drug-assisted sessions: the otherwise treatment-resistant patient who receives significant but time-limited benefit from psychedelic medicine with no opportunity for further therapeutic sessions. Such participants are not merely hypothetical, but are well-recognized by researchers in the field, and speak to an intuitive ethical problem where post-trial provisions are not available. Our position is not, however, that this is the *only* group to whom post-trial access might be owed, and to varying degrees the arguments we make will apply to other psychedelic trials, and may perhaps have implications for some non-psychedelic trials: this is a natural result of recognizing that the full context of a trial can influence what, if any, post-trial provision ought to be made available.

In the central section, we highlight three aspects of treatment with psychedelic medicines that demonstrate the atypical case they pose when it comes to provision of post-trial care: the availability of psychedelics outside of the clinical trial setting, the risk of a fixed trial end-point being an unsafe time to conclude therapy, and the idiosyncratic demands of the relationship between researcher-therapist and participant in a psychedelic trial. Having made the ethical case for post-trial provisions in psychedelic trials, we describe what forms such provisions should take. The trials themselves are different from those of typical psychiatric research, and so too are the experiences of the participants. As such, we argue that the post-trial *needs* of participants likewise vary, and while further access to the trial drug may be indicated for some, moral obligations to others may be better met by the provision of psychoeducation, further non-drug-assisted psychotherapy sessions, or lower-intensity psychological support.

Thereafter, on the basis of the normative considerations laid out, we provide preliminary suggestions about how such an expansion of support might be funded given the already resource-intensive nature of psychedelic trials. We conclude by arguing that expanding post-trial provisions in psychedelic trials, as well as meeting ethical obligations to trial participants, can serve to advance the expertise and infrastructure needed to successfully mainstream psychedelic interventions in mental healthcare, an outcome in the interests of research sponsors, researchers, and host governments.

## Psychedelic-Assisted Therapy

The classical psychedelics, a group of hallucinogenic drugs including lysergic acid diethylamide (LSD), psilocybin (the principal psychoactive component in magic mushrooms) and N,N-dimethyltryptamine (DMT, the major psychoactive molecule in ayahuasca), were first used in combination with psychotherapy in the 1950s and 60s, with the practice making a resurgence in psychiatry and psychology research today [10, 11]. The most widely used psychedelic medicine in modern clinical trials is psilocybin. Clinical trials of psilocybin-assisted therapy typically involve one to three sessions with a high dose (c. 20-35mg, see [12]) of the drug. Dosing sessions take place in the presence of one or two experienced guides, who fulfil the roles of both researcher and therapist during the trial. Dosing occurs in a relaxing, aesthetically pleasant and comfortable non-clinical environment, with participants reclining with an eye mask and headphones through which a specially designed music playlist is played throughout the session [13]. Importantly, dosing sessions are embedded within a wider program of drug-free psychological support, which aims to establish rapport with and prepare patients in advance of the drug sessions, and to support the sober reflection on and integration of material evoked by those sessions. The content and number of these post-drug sessions varies across studies, as do the psychotherapeutic models and orientations employed [14–16]. It is not yet established which psychological therapy is most effective in combination with a psychedelic for any particular indication.

A now substantial number of studies have demonstrated safety and potential efficacy of psilocybin-assisted therapy across conditions [17] including various addictions [18, 19], obsessive–compulsive disorder [20], depression [21–23], and end-of-life anxiety [24–26]. At the time of writing, there were a further 76 active and recruiting or not-yet recruiting clinical trials using psilocybin registered on clinicaltrials.gov, spanning conditions such as cluster headaches (NCT04280055,NCT02981173, depression in neurodegenerative conditions (NCT04123314; NCT04932434, and anorexia nervosa (NCT04052568; NCT04505189. There is therefore no doubt that this *“*renaissance” of psychedelic medicine is gaining traction, and has the potential to provide a welcome development in modern psychiatry and psychological therapies [10, 11].

Although the precise mechanisms of action remain to be established, both psychological and neurobiological theories offer empirically supported hypotheses for the efficacy of psychedelic-assisted psychotherapy. The psychedelic “trip” experienced during acute drug effects can generate self-insights and new perspectives, and give rise to a wealth of other personally significant and emotionally rich subjective phenomena which can be worked through safely in a supportive, carefully curated clinical context [24, 27]. At the same time, the drug induces a “hyper-plastic” brain [28–30]. It is proposed that this enhanced plasticity offers a window of opportunity to support therapeutically meaningful change in the drug-free psychotherapy sessions that follow, with focus typically on psychologically “integrating” the material that arose during the drug session with the patient’s previous narrative of their life experiences. In contrast to standard psychiatric drug trials, these psychotherapy sessions are thought to form an integral part of psychedelic treatments, as the client is supported in consolidating the insights uncovered into their conscious awareness and everyday reality in such a way as to support meaningful and sustained change in their life [18, 25, 26, 31, 32] see also [33–35].

## The Case for Post-Trial Provisions in Psychedelic Research

Here, we open the case for post-trial provisions by introducing what we take to be an intuitively strong example, though some, if not all, of the arguments for post-trial provision made in the main body of this paper generalize across a wider set of circumstances. Consider a participant in a psychedelic trial for a treatment-resistant condition. As an example, Imperial College London conducted one of the first such studies in the modern wave of psychedelic studies. This pilot study was an open-label trial of psilocybin for treatment-resistant depression, where the inclusion criteria listed “no improvement despite two adequate courses of antidepressant treatment”, though some patients had failed to achieve relief from as many as eleven treatment attempts, and all had failed to improve following CBT [21]. Although far from all participants in clinical trials of psilocybin for treatment resistant-depression had previously exhausted all guideline-adherent interventions before participation,^1^ in the first Imperial trial, consider that at least some participants had tried near enough everything that the toolbox had on offer.

Although this study found considerable success – so much so that psilocybin-assisted therapy for treatment-resistant depression has since been given a “Breakthrough Therapy” status by the FDA – it remains the case that a number of participants experienced relapse of symptoms following treatment. By the 6-month follow-up, one-third of the “treatment responders” – those who had experienced at least a 50% reduction in depression severity relative to baseline 1 week after treatment – had relapsed. This is a regrettable reality that patients can experience following any treatment for refractory depression.

However, this description of the results occludes an ethically significant dimension of the trial from the perspective of a relapsed participant. Although quantitative measures of depression severity may be the same at baseline and at six months after the trial, such a patient is not simply ‘back to how they were’ before the trial, with the impact of the trial effectively a net zero. Contrary to a trial participant’s beliefs of years or decades, and the evidence of many failed treatment attempts, they have learnt that there *is* relief from their condition, that they *can* live as others do and as they have longed to. As much as this discovery can be a blessing, it can develop into a curse should they relapse, because further sessions of psilocybin-assisted therapy are not possible, due to the thicket of restrictions preventing further administration of psilocybin outside of the legal and regulatory container of the trial.

As we see it, for further psilocybin-assisted therapy to be withheld from an otherwise treatment-resistant patient when needed again – for them to experience that an effective treatment *does* exist, but is henceforth forbidden to them – is a *harm*. More precisely, it is a *dignitary* or *emotional* harm [36, 37], a nontangible affront to a patient’s dignity or personhood in the absence of any economic loss or physical injury. Although the intangible nature of such effects makes them harder to recognise and agree on *as* harms compared to more paradigmatic examples of physical harms [38], there is evidence suggesting that patients are likely to emphasise such harms as much as physical ones [39, 40]. Given the importance placed on them, these harms warrant ethical consideration when reflecting on biomedical practice.^2^ Moreover, note that the harm in the scenario described above is only experienced *as a result of trial participation*. While it is imperative that no participant enters a trial with the *expectation* of receiving long-term benefit (as this would be a misinterpretation of the purpose of research), it is the duty of researchers and research ethics boards to ensure that harm is minimized: here the natural redress seems to be the provision of ameliorative treatment.^3^

Whereas clinical research norms may typically require only that researchers facilitate access to, or do not withhold, any normally available effective treatment to trial participants with enduring health needs following a trial, such norms fall short when considering those suffering from treatment-resistant conditions. For the patient described above, no other treatment has brought about symptom relief, but psilocybin-assisted therapy has secured clinically significant reduction or remission.

Through presenting this hypothetical case at a conference, we came to understand that this experience is in fact a reality for at least one participant from a psychedelic trial. Speaking in the same session, IR, who co-founded the Psychedelic Participant Advocacy Network (PsyPAN) related the similarity of this description to his own experience, which he recounts in box 1.

**Table:** 

I felt incredibly fortunate to be one of just 20 participants in the Imperial College psilocybin for depression pilot study in 2015. The two-dose (10mg then 25mg) trial led to a three-month period of my life where the crushing weight of depression and anxiety finally lifted after a lifetime of struggling with and being limited by it. I felt lighter within myself and the world even appeared visually brighter during this period – I finally felt free. But this afterglow gradually faded and my old thinking patterns and rumination returned during the three months that followed. I can track this descent via my emails to the Imperial team as I became increasingly desperate to cling onto this new way of thinking, feeling and being. I asked how I could best stay connected to this new state and when the next phase of the trial would take place so I could re-access the treatment that had helped me so much. The team were compassionate and responsive throughout, but they were limited in what they could do on a practical level to help. I looked into the underground but couldn’t trust I would be safe so gave up on this. I eventually ended up being referred back to my local NHS mental health services and taking largely ineffective SSRIs again. It took me four difficult years before I was fortunate enough to access the treatment again via another clinical trial (around the same time the second phase of the Imperial trial began). Finding something that worked and reduced my suffering was almost more difficult for me after the first trial, because I had no legal way to access the treatment again. It was like a bright light had briefly been shone on me but that also served to cast an even darker shadow once the benefits faded and I desperately grasped to retain them.	

### Evidence of Benefit

Even if the presented case study is accepted as demonstrating a (defeasible) responsibility to provide post-trial access, an objection may be made on clinical grounds from epistemic caution: how can we say what is best for the participant from a single trial? We don’t know yet what the best practice is after the one to three drug sessions typically employed in psychedelic trials, nor the risks of repeated drug sessions. As the effects of further drug sessions are unknown, it is better that we delay from providing post-trial access until we know more. However, the thrust of this objection seems to stem from an idealized sense of the epistemic space that clinicians standardly operate in. The result of a single trial is indeed minimal evidence compared to the multiple double-blinded placebo-controlled randomized controlled trials (RCTs) that investigational drugs must pass through before being licensed. But this focus conflates the level of evidence necessary for determining what is best for future standard practice, and that for judging what is best for a given patient. While the former requires substantial empirical grounding (e.g., successful phase 3 clinical trials), this is not true for the latter. Clinicians make treatment choices on the basis of what works, and, just as you would not continue treating a patient with a licensed drug that elicited no therapeutic response, the positive treatment response during the trial of an otherwise unresponsive patient is a highly salient data point when considering their future care. Although extensive trial data concerning risks of further psychedelic-drug administrations is not yet available, a broader body of knowledge can, and should, be drawn upon: without denying the evidentiary shortcomings of case studies in general, the careful consideration of similar cases (e.g., [41]) can nonetheless partially inform clinical judgment on a patient-by-patient basis. Alongside this weaker quality evidence, one meta-analysis suggests that trials involving multiple dosing sessions appear to support greater levels of symptom relief in depression and anxiety [42]. Large-scale population surveys of psychedelic use report no increase in adverse outcomes among psychedelic users who report multiple lifetime uses, and even suggest long-term protective effects against psychopathology [43, 44]. We also know that communities that engage in regular, ritualistic consumption of serotonergic psychedelics present no greater rates of health abnormalities than other populations [45, 46], and that in jurisdictions that permit administration of psychedelics to select patients according to case-by-case licensing, repeated sessions of psychedelic-assisted therapy can be used to positive clinical effect and without serious adverse events (e.g., [47]). With this in mind, post-trial access should be coupled with sufficiently cautious counsel from investigator teams that further sessions should be considered separate from the trial itself, are highly experimental, and as such carry non-negligible risk. So presented, providing post-trial access where clinically indicated to patients who wish to continue with treatment may represent an attractive option for ethical research conduct. As we detail below, systematizing data collection from such interventions would have significant societal benefit too, by extending clinical experience of efficacy and providing valuable additional safety data.

While the authors all feel the intuitive pull of the example case provided to justify the introduction of post-trial provision, we acknowledge that others may not, recognizing the typically higher degree of disagreement around the identification of dignitary and intangible harms compared to more typical clinical harms [38]. Instead of adding to a bioethical literature on the moral weight of dignitary harms, our argument for post-trial obligations moves from the presumptive case study outlined above, towards highlighting three atypical aspects of treatment with psychedelic medicines, which, while we do not claim to be unique within this modality, in combination demonstrate a particularly strong case:

1. ***Psychedelic access “in the wild”***: the potential for patients to access psychedelics outside of the clinical trial setting, and the risk that poses;
2. ***Challenges of short-term psychedelic therapy interventions***: the risk of trial endpoints coming too soon to end therapy safely for every participant; and
3. ***The nature of the researcher-therapist/participant relationship***: the unusual depth and intensity of the relationship between researcher-therapist and participant in psychedelic clinical trials can generate additional moral responsibilities.

## Trials Unlike Any Other: Three Reasons for Post-Trial Access in Clinical Psychedelic Research

### Psychedelic access “in the wild”

For most investigational drugs, trial participants seeking post-trial access are beholden to the drug developer (often the research sponsor) for continued access. An unlicensed drug is typically unavailable without the co-operation of those involved in its manufacture. Not so for psychedelics. Psychedelics such as psilocybin are accessible elsewhere, either in countries where they are not criminalized, or illegally obtained in countries where they are.^4^ We know that some former trial participants *do* acquire psychedelics in these ways: at least three from 20 participants in the aforementioned trial in treatment-resistant depression at Imperial College London reported as much [, p.553]^48^

Participants will be aware that psilocybin-containing mushrooms, as well as services described as psilocybin-assisted therapy, are available elsewhere: either illegally in their home jurisdiction, or legally in another country. As such, an invidious choice is imposed upon these individuals, and it is imposed upon them wholly by virtue of their participation in the trial: to passively accept a debilitating health condition which they now know can be effectively treated, or to take on significant risks in pursuit of symptom relief that they know is medically possible. To be obedient to the law, or to be well? Notwithstanding recent legislative changes in jurisdictions such as Australia, Oregon, and Colorado, psychedelic compounds are typically controlled as tightly as criminal law allows, meaning that those patients sufficiently motivated to acquire more of the drug expose themselves to legal risks inherent in criminal behavior.^5^ This is in addition to the safety risks of engaging with the illegal market, where drugs are liable to be adulterated or mis-sold. Individuals engaging an underground therapist offering psilocybin-assisted therapy enter a considerably risky space which, due to its illegality, is entirely free from regulatory oversight. Although there are knowledgeable therapists working in extralegal environments who take seriously the challenges associated with the modality [50], it is not easy for any patients – let alone particularly vulnerable or desperate patients – to discern skilled practitioners from those who are enthusiastic but under-skilled amateurs, charlatans, or worse.

As an alternative, those patients with sufficient resources may choose to undertake an international journey to a retreat in a jurisdiction where psilocybin use is not criminalized. While this may mitigate legal risk, there remain clinical and safety risks that the burgeoning retreat industry has yet to address. First and foremost, the growing “psychedelic tourism” industry is not held to the same professional clinical standards as research/therapy within the healthcare industry. Here too, there is currently no effective regulation of the quality of such entities, nor their screening, preparation and integration procedures [51]. Additionally, with growing reports of sexual abuse by shamans and guides in retreat settings, safety can be jeopardized for those contemplating a psychedelic retreat abroad [52].^6^

Although serious efforts are being made within the wider psychedelic movement to develop the therapeutic expertise, accountability, and ethical standing of non-clinical retreat centers,^7^ this does not excuse those running clinical trials from a duty to provide post-trial access where appropriate. Withholding post-trial support simply because of the existence of third-party providers in some parts of the world risks exacerbating pre-existing inequalities between participants. Even if trial participants live within jurisdictions that have permitted ‘supervised adult use’ of psychedelics, only those with sufficient means will be able to access supervised, legal psilocybin-assisted therapy or associated services with minimized risk. Other participants would still have to choose between an abiding loss of the highly valued health and quality of life benefits accrued during the trial, or taking on the legal and safety risks associated with psilocybin criminalization in order to seek out enduring benefit. As is seen in many drug policy issues [55], here too racial disparities in wealth, coupled with those in drug policing and convictions, can exacerbate inequalities. Running an ethical trial demands fair treatment of participants, including a fair distribution of burdens among them. Securing continued post-trial access, and ensuring equity of access^8^ seems to be the most appropriate response to heading off a future injustice.

### Challenges of Short-Term Psychedelic Therapy Interventions

RCTs in psychedelic therapy research tend to be short-term time limited interventions. Whilst this format may be beneficial for some patients, offering rapid symptom reduction, the optimum number of sessions for a psychedelic therapy intervention has not been studied and is to date unknown.

Given the highly individualized, and largely unpredictable subjective experiences during the acute drug effects, researcher-therapists and participants have less control over the therapeutic process than they generally might for traditional talking therapies in which therapists play a more directive role. Additionally, although the protocol-defined number of integrative psychotherapy sessions after a drug-session may be sufficient for some, perhaps even most participants, it can be insufficient for others to successfully make sense of and integrate psychedelic experiences from the trial (see, e.g., [48] p. 557, [14], p.8, [57], *passim*). These experiences, including sometimes deeply challenging, previously-unconscious, or transpersonal material, need to be assimilated into participants’ understandings of themselves, their world-view, and their everyday lives in a meaningful way. This is a novel challenge that arises as a consequence of trial participation, and it has already been acknowledged that some participants seek additional support in integration after the session(s) provided by the trial [58], p. 35,[59], p.6): i.e., it is not simply that the participants did not achieve the optimum possible benefit at the trial end date, but that the trial initiated but did not complete a process of psychological work – sometimes referred to as the *unfolding process* [35, 60] – that participants could not opt out of, but had to find external help in concluding.^9^Although the protocols and procedures of a clinical trial might impose a fixed and linear timeline, this larger therapeutic process resulting from psychedelic drug induced effects may not coincide seamlessly with the endpoint of a clinical trial. Here, a practice common to many clinical trials in which participants need support after the conclusion of the trial – i.e., referral back to existing mental healthcare support – does not ensure that the duty of care of a trial team is met. In many systems, including the UK’s NHS, demand for mental healthcare services far outstrips supply, meaning this referral can effectively mean a return to a GP. As such, the ‘existing support’ to which a trial participant is returning might be limited to those same treatments that have previously been ineffective for them. Although it is the case that participants are referred back to existing healthcare support in all trials, the uncommonness and novelty of the challenges that can arise following a psychedelic trial are such that specialist care is indicated.

The challenge of the sometimes-protracted nature of the integration process has already been recognized in some quarters. Therapists working on some psychedelic-assisted therapy clinical trials will at times make the clinical decision to refer patients for further private “integration therapy” sessions with therapists specializing in this modality because they would otherwise have been left without crucial support at the close of the trial. For the recent RCT of psychedelic-assisted therapy for major depression run at Imperial College London, aftercare was provided by an Assistant Psychologist in the form of mindfulness-based counselling to ensure there was some therapeutic containment for those who struggled with the ending or integration process [22], and this was widely taken up. The very provision of this extended therapeutic containment, alongside the breadth of participation among participants, is indicative of a shared recognition of need.

It might be objected that provision of additional drug- or non-drug sessions for some trial participants would interfere with the soundness of clinical trial data and thus be antithetical to the goals of clinical research. This need not be so, as long as the endpoints are appropriately pre-registered, and any post-trial provision within this timeframe is transparently reported. In fact, such reporting would be beneficial for designing subsequent studies and for potential clinical rollout. The specific needs of a participant cohort may be unpredictable from the outset of the trial, but that does not mean we cannot learn from outcomes for the development of subsequent studies.

Although insufficient support in this vital post-drug integration period can mean a missed opportunity for enduring benefit, the most pressing concern is the potential for the exacerbation of symptoms or iatrogenic harm. Among those for whom the trial-defined integration sessions are insufficient, we should recognize the possibility that for some this may lead to crisis [57, 64].^10^ Where this possibility might obtain, it is clinically responsible to ensure further support is provided. Psychedelic integration is a specialist competence, and not one that can be easily provided by any therapist. For those that cannot afford to access private integration therapy post-trial, if there is no infrastructure in place to facilitate at least a minimal number of private therapy sessions, there is again a tangible socio-economic barrier to harm minimization which must be addressed to uphold the ethical principle of justice.

### The Nature of the Researcher-Therapist/Participant Relationship

The potential ethical obligations of a researcher-therapist likely fall somewhere between two extremes [66]. At one limit, researcher-therapists should treat trial participants with exactly the same considerations as a therapist treats their patient during normal care. This is clearly not a viable position: researcher-therapists have a duty not just to provide care but also to generate generalizable scientific data, and consistently pursuing what is in the very best clinical interest of each participant – as if during normal care – can be incompatible with a meaningful scientific investigation, as individualized care would mean ubiquitous departures from protocol. At the very least, in an RCT, some participants have to be in the control group which may not be in those participants’ individual best interests if the treatment is subsequently found to be effective.^11^

At the other limit, the relationship could be conceived as a transactional one, in which the researcher-therapist owes the trial participant, who has freely consented to trial participation, only the care demanded by the scientific integrity of the protocol, and the correction of any harm imposed by the trial. At this limit, there is no need for researcher-therapists to provide to trial participants even any incidental prognostic or diagnostic information that arises that is well within the researcher’s expertise; a conclusion that few would agree with – indeed, clinical trial protocols implicitly reject this position when they incorporate processes for the supportive handling of incidental findings.

Rather than aligning with either position on the obligations of researcher-therapists, or any fixed intermediary point between the two, we draw from the *partial-entrustment model* of Richardson and Belsky [66], according to which, ancillary care obligations of researchers towards trial participants depend on a range of factors, including participant vulnerability (how valuable any ancillary care would be to patients), and whether there are available alternative providers of care. We have discussed above the value that our exemplar cases, treatment-resistant patients, can place on the recovery following psilocybin-assisted therapy, as well as the lack of alternatives available to them. In addition to these factors, the partial-entrustment model also points to a particular *depth* and *intensity* of the relationship between researcher and patient as more strongly grounding ancillary care obligations, and here too, there is a strong case when considering psychedelic-assisted therapy trials.

Psychedelic experiences can engender powerful, difficult, and profound states of awareness, and the competent therapeutic handling and containment of these states is seen as a critical element of clinical success [67]. As such, current trials place a significant emphasis on the “preparation” phase of psychedelic assisted therapy, which involves a substantial time spent building the therapist-participant relationship before any drug-assisted session. Psychedelic therapy emphasizes the establishment of a strong rapport with patients, to allow the securing of a sense of openness, safety, and trust.

The extremely emotionally vulnerable state that participants enter into with the researcher-therapists during treatment, which can be for prolonged periods of time (up to eight hours on a dosing day) can significantly deepen the attachment bond towards the therapist. These are often, after all, the people present during one of the most meaningful experiences of a participant’s life [25]. As such, the therapeutic relationship between a psychedelic researcher-therapist and trial participant is liable to be one of unusual *depth* and *intensity*. Since the success of the trial will involve the safe and effective administration of the therapeutic intervention, and the therapeutic intervention may be dependent on the researcher-therapist/patient relationship [13, 67], researcher-therapists will consciously *seek out* and cultivate deep and intense relationships with patients in a partially instrumental fashion, thereby increasing the weight of obligation of researcher-therapists towards their patients.

This relationship can be jeopardized when, for example, researchers shut down inquiries about where to access psychedelics to maintain or restore symptom relief, especially where reasons provided to explain this refusal are not considered compelling by participants. Researcher-therapists face an ethical double-bind when considering the prospect of directing patients to additional care elsewhere. They have an ethical responsibility to promote their patients’ autonomy and wellbeing, and discouraging someone from making their own decisions may violate this ethical principle. But the unregulated nature of underground therapy, and wider legal context, provoke concerns about the propriety (and liability) of providing information or guidance around further access.

The sense of responsibility many researcher-therapists feel towards their participants, alongside the recognition of the risk of the trial ending coming too soon to safely and constructively end the therapeutic process, has contributed to some psychedelic research centers establishing low-cost integration groups that are independent from the trials themselves. Here, participants from trials and others who have used psychedelics of their own accord can obtain harm reduction information, integrate challenging psychedelic experiences, and share their experiences with others who have been through the same.^12^ Such initiatives are set up despite the additional cost to an already resource-intensive treatment modality, and these supplementary groups are not yet funded by research sponsors.

Researcher-therapists providing long-term integration services *pro bono* (either in the context of an integration group or otherwise) in addition to their already emotionally-demanding contracted work, is a solution that is unsustainable and unjust: the needs of the many trial participants for such a service represents an obligation that is owed by all those involved in the running of the trial. It is because researcher-therapists are ‘at the coal face’ of the work of the trial, and because it is *they,* rather than the other actors of the research process, who undertake to develop obligation-inducing relationships of particular depth and intensity with patients, that they currently bear the costs of meeting participants’ post-trial needs. But given the necessity of these deep and intense therapeutic relationships for the correct implementation of the trial, the obligation to ensure that this is done safely should be distributed beyond those who are in direct contact with them, to all parties in the research process.^13^

## From Post-Trial Access to Post-Trial Care

Amid the media excitement about psychedelic drugs as a paradigm-shifting medicine, it is easy to overlook the experiential and relational quality of psychedelic-assisted psychotherapy [68, 69, p.3]. This emphasis on drug action is likely compounded by the demands of health regulators to demonstrate the safety and efficacy of *investigational drugs*, a legitimate and scientifically valuable goal. Nonetheless, the reality for patients and therapists alike is that the experiences of drug and therapy are deeply intertwined, and it is the therapeutic centrality of this experiential and relational quality which sets psychedelic clinical trials apart from other drug trials.

While much of the discourse on post-trial access for standard pharmacology trials focuses on access to the investigational medicine itself,^14^ we propose that post-trial provision in trials of psychedelic-assisted psychotherapy needs to be seen more broadly. We acknowledge that research cultures can take a long time to change, and as such it may be some time before post-trial *access* is practicable. However, the broader perspective of *post-trial care* provides an opportunity for smaller, more incremental steps that can be enacted sooner and that will support the development of the wider psychedelic eco-system that will, post-licensing, be able to responsively meet the diverse and individual needs of patients. Over and above explicit provision of additional psychedelic-assisted therapy sessions where clinically indicated, this ongoing support might include basic psychoeducation and harm reduction resources about mental health or psychedelic use, researcher-affiliated community-based initiatives and peer support groups, and short or longer-term (individual or group) counselling and psychotherapy. Importantly, such provision will not only service the needs of future study participants, but will also be beneficial towards the generation of a safe eco-system for the clinical roll-out of psychedelic medicine. Importantly, conceptualizing post-trial provisions as *care* in this way also creates a framework by which provision need not fall solely on the researcher-therapists carrying out clinical trials, but rather invites the wider community, and in some cases the participants themselves, to take an active role in developing this supportive infrastructure.

## Post-Trial Provision and the Future of Psychedelic Medicine

Independently of the strength of the ethical case for the provision of post-trial provision following psychedelic trials, reservations may still be voiced that the provision *any* post-trial care represents an unrealistic ideal, not only in terms of resource demand (especially for university-driven, rather than commercial, research^15^), but it may elide an important distinction, which should be recognized and maintained, between research and healthcare. In this view, our calls cast the obligations of researcher-therapists as too close to those of therapists delivering normal care. Access to any intervention or support beyond the confines of a trial is, properly speaking, healthcare, and as such is beyond the specific mandate of research sponsors, who operate with finite funds. Moreover, some funders, such as the U.S. National Institutes of Health, are not authorized to fund normal healthcare services if requested as expenses by grantees.

Such objections underline the shortcomings in the status quo, which we propose could be profitably addressed to the benefit of the medium- and long-term future of psychedelic medicine. For reasons outlined above, the provision of long-term integration groups, or further psilocybin sessions, following the trial, is *not* straightforwardly normal healthcare. Both ‘booster’ psychedelic sessions, and long-term integration services, will likely feature as a standard component of good psychedelic healthcare, and as such will form part of a *future* well-functioning psychedelic healthcare system. But neither of these components is available *now*: despite the recognition of their importance for at least some participants [33, 47, 70–72], there are no means for participants to secure them in the healthcare system as it exists at present; there is not yet a wider psychedelic medicine ecosystem that can attend to these needs to direct participants towards.

Between the status quo, and a well-functioning psychedelic healthcare system, technical and physical infrastructure must be expanded, requiring the growth of knowledge and practical systems in just the areas in which we are advocating for the expansion of post-trial provision. To that end, we invite the various partners to a psychedelic research study to conceive of post-trial provision not simply as an expensive duty to be discharged, but rather as *an integral site of psychedelic research*, and an appropriate destination for research funding. Even the studies investigating compounds that will not be licensed for 5–10 years provide opportunities to understand best practice for psychedelic treatment and aftercare *now*. By building aftercare into the clinical trial framework, including appropriate reporting and monitoring of practice and outcomes, our understanding of the efficacy of post-trial provision outside of the parameters of a given trial can be developed in advance of the licensing and wider roll-out of these medicines.^16^

In the same vein, increasing funding for long-term integration services offers an opportunity for long-term post-trial surveillance to detect treatment-related harms, and psychological challenges that may arise from a treatment modality that involves meaning-shaping experiences. Such data will ultimately pay dividends to research sponsors and the project of psychedelic medicine, as will the expertise-development pertaining to how to design and implement integration groups and practices to optimize the longevity of positive clinical outcomes.

## Meeting the Challenge of Post-Trial Care: Potential Concrete Steps for Stakeholders Named in the Declaration of Helsinki

### Governments

Although national governments operate at a distance from the psychedelic clinical research process, there is a significant facilitative role they can play. Since a swathe of the challenges related to psychedelic research stem, directly or indirectly, from the controlled status of the drugs under investigation, revision of this status can significantly contribute to alleviating the challenges. In the UK, the Schedule 1, Class A status of psilocybin simultaneously introduces financial and bureaucratic burdens that contribute to the feasibility of providing post-trial access, discourages researcher-therapists from offering harm-reduction advice to those resolute in their determination to seek out further psilocybin after the trial, and imposes the risk of a criminal record upon people seeking what may be the only effective treatment for otherwise refractory conditions. Removing criminal penalties for the possession of psilocybin would reduce the legal risks imposed on former trial participants, and need not be cast as some bold or experimental new practice in drug policy: one approach would be the decriminalization of psilocybin, which would return its legal status to that of 2005, until when psilocybin mushrooms were legal to supply and possess in the UK. Despite ample wider arguments for decriminalization [55, 74], such a move would not remove the safety risks associated with former participants seeking post-trial treatment from unregulated spaces. Instead, revising psilocybin’s schedule downwards to Schedule 2 (where significantly more harmful drugs such as heroin and cocaine sit), or to a bespoke schedule to permit research and post-trial access but not broader prescription, would meaningfully reduce the cost of the main trial, and of post-trial provision, making these provisions more easily realizable by sponsors. Such a rescheduling, achievable through statutory instrument, would require comparatively little legislative time, and need not be presented as a radical move: the strength of the scientific grounds for rescheduling psilocybin has been well rehearsed [75], and is a move with significant public support in the UK [76].

### Investigator Teams and Researcher-Therapists

As described above, the idiosyncratic nature of the role of researcher-therapist in a psychedelic trial generates a thicket of competing moral demands: a recognition of enduring support needs; the risk of the trial ending before the therapeutic process can be safely ended; earnest requests for further, clinically-indicated drug-assisted treatment; information-seeking about alternative sources of psilocybin from trial participants to whom there may be a sense of indebtedness, set against the clear restrictions of criminal law and/or institutional liability guidelines; as well as the non-negligible risks associated with treatment-seeking in unregulated spaces. Some researcher-therapists have sought to resolve this conflict with *pro-bono* work supporting longer-term integration groups: a laudable initiative which nonetheless demands significant additional emotional labor. As we suggest above, such initiatives ought to be properly funded and staffed, an aim that relies on buy-in from research sponsors who may be unfamiliar with the unusual features of psychedelic-assisted therapy as a treatment paradigm.

So long as the status quo renders post-trial access infeasible, researcher-therapists will be faced with the challenge of responding to trial participants who inquire about further drug sessions. As previously discussed, we know that some trial participants seek out further treatment elsewhere. However, such participants may not be cognizant of the absence of regulatory oversight of underground therapy to ensure that good practice is upheld, or that the experience of underground therapy may be vastly different to that received in a clinical trial setting. Even where a researcher-therapist judges that further drug treatment may be in a participant’s best interests, the legal and liability implications of discussing illegal activity while representing a research institution, and the risks associated with unsupervised treatment or unregulated therapy, can incline practitioners to discourage participants or shut down these conversations. As such, we invite readers to consider the (understandable) choice to rebuff such inquiries as structurally equivalent to abstinence-only sex education. While there is no ‘safe guidance’ that will totally eliminate risk, for reasons rehearsed above, the risks of providing harm reduction advice should be weighed up against the risks of inaction. Despite the incipient nature of the field, much is known, and can be shared, about good practice regarding dosage, set and setting, while trial participants have first-hand experience of the importance of psychological and physical safety in psychedelic therapy. Developing standardized procedures or resources for providing such advice where needed, and building this in to research protocols, will help ease the anxiety felt by research-therapists who find themselves in this potentially challenging position. Pilecki et al*.* [51] present guidelines for therapists to provide preparation and integration information within a harm-reduction framework, without being complicit in delivering psychedelic therapy itself.

Including such psychoeducational harm-reduction contingencies into trial protocols may also act as a first step towards normalizing post-trial care. The more researchers include post-trial provisions in conversations with funding bodies, sponsors, and research ethics boards, the more it will become a zeitgeist of the modern era of psychedelic clinical trials.

### Research Sponsors

For research sponsors, upon whom much of the costs of expanding post-trial care provision will ultimately fall, we are conscious that the idea of such expansion may seem to be an expensive ethics checkbox to be filled.^17^ Rather than pointing to the formal ethical mandate for providing such care that can be found in the Declaration of Helsinki, we invite such actors to consider robust post-trial provisions as a future-proofing investment for their longer-term business viability. By focusing ahead, beyond simply meeting clinically significant endpoints in a trial, providing post-trial care *now* can support the development of the infrastructure necessary for a psychedelic medicine ecosystem in future. By holding a wider perception of post-trial care as extending beyond post-trial access, and treating this provision as a site of research, we will thereby increase our understanding of the best, most cost-effective practice for repeated drug sessions, minimizing relapse, and providing appropriate long-term support to those who need it.^18^ Currently, time spent on post-drug integration within the framework of trials is minimal, leading to a lack of data on best practice for “good integration” [58]. We know that participants feel it important to be able to talk through and about their psychedelic experiences with others who have been through the same, long after the conclusion of a trial [48]. Seeking out ways to reduce the resource-intensiveness of integration, for example by investigating how much of the process can be peer-led or co-facilitated, can further enrich our understanding of this as-yet under-theorized process [34].

### Other Stakeholders

Our presented suggestions for the three stakeholders named in the Declaration of Helsinki – researchers, host governments, and sponsors – do not preclude the possibility that other actors could have a role to play in the realization of a system of meaningful post-trial care within the contexts of psychedelic trials. Institutional Review Boards, which provide a final ‘green light’ to clinical trials, might consider requiring substantial information from prospective researchers about provisions for post-trial care on the basis of the considerations outlined in this paper. However, considering the substantive clinical and normative novelty related to the biomedical use of psychedelics, it may be the case that some boards are unfamiliar with the relevant differences to standard psychopharmacological trials. As such, it is incumbent on the research community to support boards’ decision-making by providing impartial information about the special considerations relating to psychedelics. In addition, we do not immediately rule out the prospect that the costs of post-trial care might be at least partially supported by trial participants themselves: in other research contexts, co-payments and sliding scale drug fund programmes have been deployed in efforts to balance the ethical obligation to provide for post-trial access without disincentivising research through additional excessive costs [77], see also [78, 79].

## Moving Forward

In the foregoing, we have argued that for psychedelic clinical research trials to act in a Helsinki Declaration-compliant manner, and in order to ensure robust standards of ethical practice, the field should seek to broaden the current practice of post-trial care. Precisely how the boundaries for appropriate post-trial support should be redrawn remains a matter for considerably more discussion: as with any shift from the status quo, such a move naturally raises questions about implementation, from ethical, practical, and resource-constraint perspectives. It may be that running a psychedelic trial for treatment-resistant depression incurs greater post-trial obligations towards participants than does a trial for tobacco cessation, and it may be that obligations towards a participant who has tried and failed to gain symptom relief from nine established treatments will differ in weight and shape to obligations towards a participant who had only tried two established treatments before trial participation. Further work is required to articulate what principles, or thresholds, might usefully be applied to translate the recognition of a duty towards (some) participants into implementable guidelines about quite how best to discharge that duty. Provision might sometimes be the sharing of harm reduction advice, sometimes the provision of long-term integrative counselling, and sometimes access to further psychedelic-assisted therapy sessions. Regrettably, interweaving practical realities – particularly guidelines governing research funding and national laws restricting the legitimate use of controlled drugs – present challenges to moving away from the status quo, culminating in a moral tragedy: despite the strength of the moral case for trial participants to be provided with extended care where needed following the conclusion of a psychedelic trial, there is no definitively compelling reason for any given actor to be responsible for discharging this obligation. There are considerable costs associated with the provisions we are endorsing, and these costs must be borne somewhere. We take the responsibility to be diffuse, requiring each of the actors called upon in the Declaration of Helsinki – researchers, research sponsors, and host governments – to work in concert for the advancement of practice, which will serve not just to better meet moral obligations towards trial participants today, but will also support the development of infrastructure and expertise needed for the psychedelic medicine ecosystem of tomorrow.

## Data Availability

Data sharing is not applicable to this article as no new data were created or analyzed in this study.

## Footnotes

1 Here we acknowledge that the practical and ethical challenges posed by our example case may vary according to *how many* guideline-adherent therapies had been tried before trial participation. The weight and nature of obligation towards participants successfully treated in a trial of psilocybin-assisted therapy may differ when a participant has previously tried two, rather than eleven, recognised therapies.

2 Avoidance of harms speaks to the clinician’s duty of *nonmaleficence*. We suggest that the patient experience described in this scenario, if not judged by the reader to be properly classified as *harmful*, is set within the context of the withholding of a significant benefit, with the concomitant implications for the duty towards *beneficence*.

3 Such provision is consistent with Principle 15 of the Declaration of Helsinki: Appropriate compensation and treatment for subjects who are harmed as a result of participating in research must be ensured.

4 While the current article focuses on psilocybin-assisted psychotherapy, the arguments apply equally to the functionally similar psychedelics such as LSD, and to a lesser extent to ketamine- and MDMA-assisted psychotherapy, which are understood to work through different mechanisms, but can employ a similar psychotherapeutic approach and are alike in the relevant aspects. The argument centred on the illicit availability of an otherwise investigational drug also bears upon clinical trials of cannabis-based medicines.

5 Importantly, these drugs are *not* habit-forming [49], and, despite the criminality involved here, the desire to undertake more drug experiences is best understood not as a manifestation of addictive craving, but rather to provide continued successful relief of distressing and/or otherwise treatment-resistant symptoms.

6 We do not seek to claim that there are no instances of grave sexual misconduct in clinical settings [53], including in contexts involving psychedelics [54]. But whereas the prospect of violations within such settings are within the power of clinical teams to address, this is not true where participants needing additional support feel compelled to seek it in spaces outside of regulatory oversight and accountability.

7 See, for example, the work of Guild of Guides in the Netherlands and the International Center for Ethnobotanical Education, Research, and Service (ICEERS).

8 Here, we use the term *equity* to refer, not to the equal distribution of resources, but *needs based* distribution. This may entail the provision of additional resources to meet the needs of disadvantaged participants both within the trial, and in post-trial care [56].

9 Consider also, that significant numbers of those who undergo psychedelic experiences outside of trial settings can have extended difficulties thereafter, with many seeking out psychological support afterwards (see [61–63])

10 While this may be more frequently the case for psychedelic interventions practised ‘underground’ [65], where there is greater variability in practitioner expertise, and more complex patients who would be excluded from clinical trials access drugs, the reality that psychedelic experiences have the potential to engender crisis is sufficient to warrant contingency planning in trials.

11 Even in studies employing cross-over designs, it is still not in the best medical interests of half of the participants to wait months before receiving effective treatment.

12 At Imperial College London, for example, these groups are made free to study participants – a gesture currently made possible by the good will of those running the group, rather than explicit support from the research sponsor. Importantly, although these spaces are often held by therapists, they are not therapy sessions, but are more akin to peer support groups.

13 Consider the structurally similar phenomenon of teachers in underfunded schools feeling compelled to purchase school supplies from their own salaries. Although teachers are the proximate actors embedded in a relational context which may generate obligations towards schoolchildren, teachers do this work on behalf of a range of stakeholders, and it is appropriate that the costs of the task of teaching are divided fairly. Here, we agree with Millum’s ([4], p.150) argument that the responsibility for post-trial provision is distributed across stakeholders beyond the investigator team: ““If I hire you to do something which will predictably lead to you acquiring costly responsibilities, a fair pay deal should include the costs of fulfilling those responsibilities””

14 However, this is an area of active contention, with provision of access to “other appropriate care or benefits” included in the text of the 2004 and 2008 versions of the Declaration of Helsinki, but dropped from its 2013 iteration.

15 An ostensibly ethical grounds for rejecting our suggestions, driven by the resources that they demand, is that, by expanding post-trial provisions and thereby increasing the costs of the trial, the threshold for securing sufficient grant money is raised, and fewer trials will be able to proceed. As such, the progress of scientific medicine will be impeded, and fewer participants will be able to access a potentially helpful medicine. It may well be the case that the extra costs associated with our suggestions represent a fatal expense for the economic viability of some trials. But we invite readers to remain open to the possibility that the status quo balance between speed of scientific progress and robustly ethical treatment of participants might not be the optimum one.

16 The concern that provision of post-trial access, and other post-trial care, may detract from the scientific value of data collected from a trial, is a reasonable one, but not, in our view, one that cannot be accommodated. We are not here arguing for the provision of additional interventions before the trial’s primary endpoint (typically symptom reduction at 3 or 6 months) has been reached. Providing post-*trial* care following this endpoint will make interpretation of (very-)long term follow-up data more challenging. In line with Richardson et al. [73] we recommend advance planning to determine if compromise solutions can meet both the study’s scientific aims and additional care responsibilities. We also note that, by following outcomes where post-trial care is provided, a valuable new line of complementary, ‘real-world’ data is generated.

17 This is particularly understandable on the part of established pharmaceutical firms entering the psychedelic space, for whom the focus on a relational dimension to a clinical trial may be an unrecognisable feature.

18 Although space constraints limit exploration of how sponsors might discharge this responsibility, see Evans [65], who proposes that commercial operators in the field invest at least 1% of their capital to integration research and services, outlining a number of forms that this might take.
